# 1784. Appropriateness of Intravenous Vancomycin in a Canadian Acute Care Hospital

**DOI:** 10.1093/ofid/ofac492.1414

**Published:** 2022-12-15

**Authors:** Heather Glassman, Arif Ismail, Stephanie W Smith, Jackson J Stewart, Cecilia Lau, Dima Kabbani, Justin Z Chen

**Affiliations:** University of Alberta, Edmonton, Alberta, Canada; University of Alberta, Edmonton, Alberta, Canada; University of Alberta, Edmonton, Alberta, Canada; Alberta Health Services, Edmonton, Alberta, Canada; Alberta Health Services, Edmonton, Alberta, Canada; University of Alberta, Edmonton, Alberta, Canada; University of Alberta, Edmonton, Alberta, Canada

## Abstract

**Background:**

Intravenous (IV) vancomycin is a commonly prescribed antimicrobial. However, there is limited literature assessing IV vancomycin appropriateness. The objective of the study was to assess the appropriateness of IV vancomycin prescriptions in a Canadian acute care hospital.

**Methods:**

Prospective audit and feedback (PAF) was conducted on all new IV vancomycin prescriptions in hospitalized adults (age ≥ 18 years) at the University of Alberta Hospital from January 17 to February 11, 2022. Appropriateness was assessed against institutional prescribing guidelines (Bugs & Drugs® and Alberta Health Services Formulary Prescribing Guidelines). Verbal and written feedback were provided to the attending teams.

**Results:**

A total of 109 prescriptions were audited. Median age was 57 (IQR 43-72) years and 42% were female. 65 (60%) were admitted to Medicine, 18 (17%) to Surgery, and 26 (24%) to Intensive Care units. MRSA colonization was present in 32 (29%) patients, β-lactam allergy recorded in 21 (19%), and acute kidney injury (AKI) in 21 (19%). Infectious Diseases consultation (IDC) occurred in 29 (27%). The top indications were skin and soft tissue, pulmonary, and bloodstream infections (Table 1); 70% of prescriptions were empiric in nature.

Overall, 43 (39%) prescriptions were assessed to be suboptimal. Antimicrobial Stewardship program (ASP) recommendations were made in 56 prescriptions, totaling 63 unique recommendations. Vancomycin was recommended to be discontinued in 24 (43%) cases or changed to a different agent in 15 (27%). Regimen optimization (duration or frequency change) was recommended in 4 (7%). ASP recommended investigations in 8 cases and IDC in 12. Full or partial acceptance was achieved in 49 cases (88%).

IDC was associated with greater appropriateness (83% vs 53%, p=0.004) as was MRSA colonization (75% vs 55%, p=0.047), but AKI was not (62% vs 60%, p=0.888). Adjusting for age, AKI, and MRSA colonization, IDC remained a significant predictor of vancomycin appropriateness (OR=4.27, [95%CI 1.44-12.70]; p=0.009).

**Figure:**
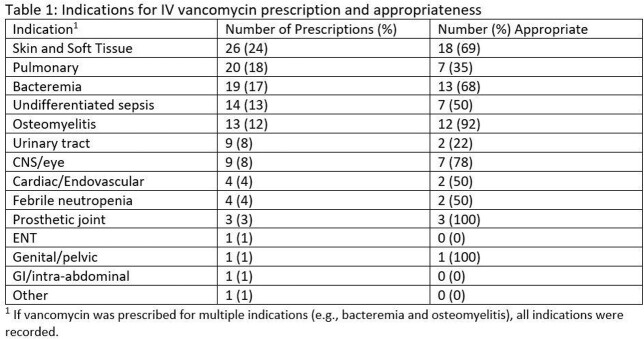

**Conclusion:**

IV vancomycin prescriptions were suboptimal in 39% of cases. IDC was associated with increased appropriateness. This pilot informs the need for antimicrobial stewardship intervention and the importance of IDC at our center.

**Disclosures:**

**Dima Kabbani, MD, MSc**, AVIR Pharma: Grant/Research Support|AVIR Pharma: Honoraria|GSK: Honoraria|Merck: Grant/Research Support.

